# A Community Empowerment Framework to Promote Social Connection and Engagement of South Sudanese Youth in Australia

**DOI:** 10.3390/ijerph22060865

**Published:** 2025-05-31

**Authors:** Troy Pittaway, Elisha Riggs, Jaya A. R. Dantas

**Affiliations:** 1 Curtin School of Population Health, Faculty of Health Sciences, Curtin University, Perth, WA 6102, Australia; troy.pittaway@vu.edu.au; 2 Intergenerational Health, Murdoch Children’s Research Institute, Melbourne, VIC 3052, Australia; elisha.riggs@mcri.edu.au; 3 Department of General Practice, The University of Melbourne, Melbourne, VIC 3010, Australia

**Keywords:** intergenerational conflict, South Sudanese, youth, empowerment, wellbeing, community engagement

## Abstract

**Background:** Significant factors impact the wellbeing of South Sudanese youth who settle in Australia. This article proposes a community empowerment framework based on outcomes from research and community feedback undertaken in Melbourne, with South Sudanese youth and elders to improve social connection and community engagement of the youth. **Methods:** Twenty-three semi-structured interviews, four focus groups and two community forums were conducted using qualitative, case study methodology. Data were collected from South Sudanese youth aged 14 to 21 years, social workers, elders and parents from the South Sudanese community. Inductive thematic analysis was used to gain an understanding of the social issues facing South Sudanese youth, **Results:** Four themes—health and wellbeing, the experience of racism, sports and intergenerational conflict—were identified during analysis. These themes were then used to develop and propose a Community Empowerment Framework (CEF), that outlines strategies to empower South Sudanese youth to improve their wellbeing at different levels: personal empowerment, career empowerment and community empowerment (both internal and external). **Conclusions:** The CEF provides a community-informed model for service providers and policy makers to promote positive community engagement and social connection to improve the lives of South Sudanese youth living in Australia.

## 1. Introduction

Forced migration continues to be a global concern. At the end of 2022, over 108.4 million were forced to be displaced due to unprecedented circumstances including conflict, persecution, human rights violations and events that disturbed public order [1]. Australia offers humanitarian settlement through its migration program and resettles around 13,000 each year, with an increase to 20,000 places for 2023–2024 [2]. Between 1995 and 2015, over 3000 South Sudanese were resettled in Australia due to the ongoing war and conflict in South Sudan [3]. According to the 2021 Census data, there are 8255 South Sudanese residing in Australia [4].

South Sudan is a multilayered and multicultural country comprising different ethnic groups known as tribes, with different languages, religions and cultural heritage [5]. Despite the many differences, the South Sudanese united to confront the British-appointed Sudanese government which was made up of predominantly Islamic Arabic Sudanese, who had dominion over the country of Sudan and the South Sudanese peoples. After many years of war with Northern Sudan due to complex political, ethnic and religious conflicts that were in large part a result of the destabilizing force of British colonization, South Sudan became an independent nation in 2011, to great optimism of the South Sudanese people.

Despite this optimism, by 2013, instability within the newly formed government of South Sudan was apparent, and historical conflicts fueled by tribal and clan tensions resulted in the breakdown of this newly formed institution—starting a new civil war [5,6]. The ongoing conflict reflects a country many of the citizens of South Sudan have fled looking for safety from a volatile and protracted conflict. It is in this context that many South Sudanese have arrived in Australia as refugees, seeking safety away from the war and conflict of their homeland. Forced migration creates many issues for people who have fled South Sudan, including navigating the settlement issues and the nuances of new culture and community expectations.

Predominant of the South Sudanese population residing in Victoria, Australia consists of young people, with 62.4% of the population being under the age of 25 [7]. South Sudanese youths faced a unique and potentially burdensome existence when growing up in the Australian culture whilst also attempting to meet their parents’ expectations of maintaining a connection to their South Sudanese culture [8,9]. Whilst South Sudanese community-specific suicide rates were not formally documented, community group leaders have voiced their concerns on the observed overwhelming suicide incidents among South Sudanese youths [10,11]. Australian Institute of Health and Welfare has also reported that migrants with refugee backgrounds were found to be 1.7 times more at risk of suicide compared to ‘other permanent migrants’ [12]. Hence, refugee-background youths are an at-risk population, and in Australia, there is a lack of research informing the wellbeing of South Sudanese young people [13].

Previous research and the works of the first author have found that South Sudanese youths have experienced mental health challenges, such as anxiety, depression and post-traumatic stress symptoms due to the war in their home country [14,15]. In addition to the direct or indirect trauma that South Sudanese youths faced due to the ongoing conflict in South Sudan, they have also encountered familial and environmental stressors that further negatively impact their mental health [14]. From a family perspective, South Sudanese youths are likely to experience intergenerational conflicts due to the differing cultural norms of their home country and settlement countries, which causes tension between youths and the older generations [16,17]. From an environmental or external perspective, South Sudanese youths also experienced racism and marginalization from different parties and mediums, which includes negative interactions with police, racial profiling in the media and alienation from the broader society [9,18]. These compounded experiences have impacted South Sudanese’s mental health, resulting in the use of various coping mechanisms, where some are adaptive such as sports [19,20], and maladaptive such as cannabis use [14].

There are many frameworks developed to improve the wellbeing of vulnerable and at-risk communities, such as refugee communities. One of the frameworks is the Psychosocial Conceptual Framework, which examines social ecology, human capacity and culture and values in post-conflict communities [21]. The framework focused on the community’s existing personal resources, as well as available Physical, Economic and Environmental resources to empower the community, improving their wellbeing [21,22]. Another framework that was developed was the Transformative Psychosocial Framework for adult refugees settling in Australia [23]. The framework expanded on the role of personal agencies (identifying, assessing and transforming personal constraints) and social structural affordances (past refugee life experiences, encountering social cultural dissonance, confronting racism and discrimination and facing employment challenges) in promoting wellbeing in the community [23]. Though both frameworks were able to provide an overarching guideline for community support workers to empower the South Sudanese youths and their families, both frameworks lack tangible strategies that are crucial in supporting South Sudanese youths within their family system and strategies that support empowerment within a youth population. This paper posits to develop a community framework specific to South Sudanese youths.

## 2. Materials and Methods

### 2.1. Study Design

Using an exploratory, qualitative case study approach in a real-world setting, the intent of this study was to give a ‘voice’ to South Sudanese youth living in southeast Melbourne, Australia. The case study methodology was selected as its main characteristics help convey the realities of participants’ experiences to the reader and is a method that can be widely employed across many disciplines—such as the wellbeing of South Sudanese youth [24]. The key components of a case study approach include tapping into the viewpoints of participants and allowing participants to describe their experiences in their own words. It is an ideal methodology when there is a need to ‘closely examine contemporary events’ and in the context of this study, South Sudanese youth shared their experiences of living in Melbourne, Australia, and the impact it has on their health and wellbeing [25].

An exploratory, qualitative case study approach was used for the larger study. A case study approach is an ’empirical method that investigates a contemporary phenomenon (‘the case’) in-depth, and within its real-world context‘ [26]. A case is a situation, or unit, such as a person, a family, an organization or a site which could be perceived as micro (individual), meso (an organization—schools and workplaces) or macro (community in Melbourne) [27]. The qualitative case study research design permitted a rich understanding and in-depth exploration of culture and the multiple complex issues experienced by the participants [28].

### 2.2. The Psychosocial Conceptual Framework

**Figure 1:**
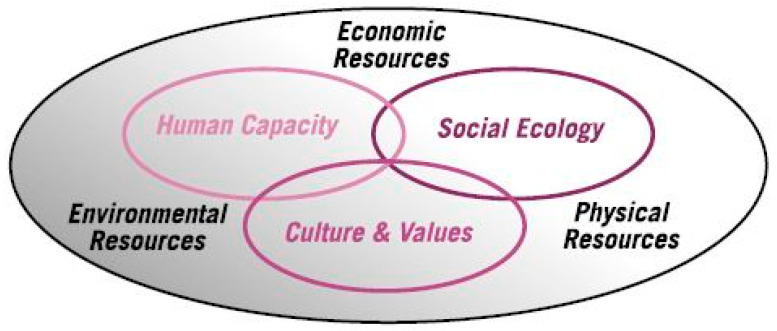
Psychosocial Conceptual Framework.

Drawing on previous research [17], this study used the Psychosocial Conceptual Framework, outlined in Figure 1. The Psychosocial Conceptual Framework underpinned the study and the analysis of data from the case studies, as it allowed for themes to be explored through a holistic lens. The three core domains of the psychosocial model are (1) Human Capacity, (2) Culture and Values and (3) Social Ecology—together with the available Physical, Economic and Environmental resources. This framework has been used extensively since it was first proposed and is used for post-conflict settings or with refugee and vulnerable populations. These domains map the human (physical and mental wellbeing, the skills of people and their livelihoods), social (relations within families, links with peer groups, religious, cultural civic and political institutions) and cultural capital (cultural values, may include religious and other traditions that stabilize and lend resilience to communities) available to people [21,22].

### 2.3. Study Setting

The study is conducted in Melbourne, the capital city of Victoria and the second-largest city in Australia [29]. Melbourne has an estimated resident population of 117,936 and a population density of 4752 persons per square km [30]. There are around 13,532 individuals with South Sundanese ancestry residing in the state of Victoria, and 3121 were born in South Sudan [7]. Most of the South Sudanese communities are young in age, with the largest cohort aged 0–14 (40.2%) [7]. From 2001 to 2010, most South Sudanese people came to Victoria through the Australian Government’s Humanitarian Migration Program [7].

The study setting was a community outreach center that was frequented by South Sudanese young people to attend events and activities. The center is in southeast Melbourne, a culturally diverse area characterized by high refugee settlement. All recruitment of participants for interviews, focus group discussions and community forums, took place at a community center. The first author was employed for over a decade as the community services manager at this outreach center and had worked extensively within the South Sudanese community. Through this role, he had developed strong connections with the community. His work involved engaging with both young people and their parents which enabled access to the community due to a pre-existing known and trusted relationship.

### 2.4. Community Support for the Research

The first author approached community elders to discuss the study and sought their support at different stages of the project, it was important to do this from a cultural perspective. For instance, the parents of participants under 18 years were approached by the first author, along with a trusted community elder to discuss the research and parental or guardian consent was obtained. Ethics approval was obtained from the Human Research Ethics Committee at Curtin University (approval HR 139/2012).

### 2.5. Participant Recruitment

Purposive and snowball sampling [31] was undertaken at the community center where the first author worked. Participant recruitment continued until data saturation was met. We have perceived data saturation when themes that emerged were repetitive and there was no new information being spoken about [31,32]. There were 16 male and seven female youth participants, aged between 14 and 21 years who took part in interviews between 2013 and 2017.

All participants spoke English and were provided with a plain-language summary explaining the study, were given the opportunity to ask questions about their participation, and subsequently signed a consent form. Participants understood that they could withdraw from the study at any time; however, the researchers were aware that this is more difficult with focus groups. A protocol was established to support upset or distressed participants whereby interviews were discontinued, and support was provided as appropriate. It was made clear to participants that their decision to not participate would not affect their access to services and activities.

### 2.6. The Interviews

An interview schedule of 15 open-ended questions was developed by the researcher in consultation with a youth social worker who worked with the South Sudanese community. Two South Sudanese elders were engaged to confirm the appropriateness of the questions and helped to finalize the questions. The questions explored participants’ migration history, experiences living in Australia, family dynamics, self-reported mental health and coping strategies. As all the participants had a high level of English language proficiency, the interviews were conducted in English without the need for an interpreter. Twenty-three semi-structured interviewees were conducted. Quotes from the interviews are presented with pseudonyms.

### 2.7. Focus Groups Discussions

There were two focus groups for youths, one for women (*n* = 7) and one for men (*n* = 16), and they were the participants that took part in the interview. Focus groups were held to encourage community engagement from the participants, and it was observed that participants were more willing to share their thoughts in a group setting rather than an interview setting. A third focus group comprised of eight parents (4 female, 4 male) and three elders (1 female, 2 male). Participants were invited through connections at the community centre. In total, there were 23 youths, eight parents and three elders who participated in focus groups. A final fourth focus group involving five community elders and an interview with a local youth worker who worked with South Sudanese youth was conducted to present the results and confirm and validate the themes that emerged from the research.

### 2.8. Data Analysis

The interviews and focus groups were audio recorded and transcribed verbatim by the first author. Using the thematic analysis process described by Braun and Clarke [33], an inductive approach was used to identify and examine underlying ideas and concepts to inform the meanings of the interview data [33]. Analysis commenced with reading and immersion of the interview and focus group transcripts. The transcripts were read several times and notes were made. Codes were generated by ascribing meanings identified from the interviews [34]. Nvivo software (Version 11) was used to code the data for thematic analysis. The four key themes identified were as follows: (1) experiences of racism; (2) a sense of belonging; (3) sport and wellbeing and (4) intergenerational conflict. The themes were then mapped to the domains of the Psychosocial Framework (see Figure 1).

### 2.9. Rigour and Community Forums

Rigor was determined by the four criteria ’credibility, confirmability, dependability and transferability‘ which were critical vis-à-vis the depth of the data collected in this study rather than the sample size of the interviews [35]. To enhance the credibility and overall trustworthiness of this research, systematic checking, ongoing interpretation of data and an audit trail were utilized to ensure information relating to the study design, methods and analysis were transparent and could be replicated. Member checking, the process of sharing interview findings with the community to ensure data accuracy, was undertaken through community forums to validate results and themes and enhance the trustworthiness of the data [36]. More details on the rigour of this research study can be found in our previous publications [9,14,17,20].

Two community forums were held to share and confirm the findings from the study. Various members of the South Sudanese community including parents, elders, youth and other stakeholders attended. The first forum had 65 attendees and the second 47 attendees. Community members were invited to share their reflections which were then incorporated into the recommendations and the final Community Empowerment Framework.

## 3. Results

The results section first summarized the themes that South Sundanese youths have experienced. Later, the community empowerment framework that was developed as a result of these themes will be discussed.

### 3.1. Experience of Racism

The issue of racism was a constant concern amongst the participants, particularly how they are portrayed by the media and the subsequent political rhetoric and police interactions. Many participants recounted stories of walking with their friends, who were often South Sudanese, to and from home and the local basketball courts, but would be stopped by police inquiring who they were and what they were doing. This would create a great deal of frustration for the young people which often escalated, and the young people being threatened or physically pushed or grabbed by the police. Often, the interactions with the police would be intensified when they travelled on public transport. Participants recalled multiple accounts of police stopping them and their peers and demanding to look in their bags, without justification. The inequity of this was highlighted when they saw that their White friends were not subject to a similar level of inquiry.

*“We are easy targets for the police because of our skin colour and what the media say about us, so they come after us when they see us out. I wish they punished you for the right reason, like if I did something wrong, not just because you are Sudanese and you are trying to get home from your friend’s home and they just hit you and grab you and the reason they tell you they did this is because you were screaming and aggressive towards them, even when that was a lie. I screamed and became angry after they hit and grab us for no reason”.*

*(Shudier, female youth)*

### 3.2. A Sense of Belonging

Media coverage and populist reports by Australian politicians increasingly malign the South Sudanese community and participants reported that this was felt deeply by them and diminished their sense of belonging within the Australian community.

*“The hardest thing is that some people are racist. They think that all Sudanese are the same. They see us on the news and think we are all bad. Like if white people do something bad its nothing, its normal, but if its Sudanese its always on the news. If its white people they say nothing but if it’s Sudanese it’s there in full detail”.*

*(Giit, male youth)*

The resignation that many felt from being an outsider led to many no longer trying to ‘fit in’ to the Australian community.

### 3.3. Sport and Wellbeing

A common coping strategy used by the participants was to play sports or be involved with art and music activities. Participants reported that these outlets helped them to deal with feelings of isolation, depression and anxiety. Participants explained that having a space for young people to feel connected to others and validated them as individuals (as opposed to a stereotype) was important to their mental health and wellbeing. Sport also offered a therapeutic outlet promoting relaxation and time to self.

*“When life gets to stressful man, I just go to the park and play basketball and just shoot [goals] by myself. I don’t talk to anyone, I just be quiet, listening to the ball bounce, concentrating on the game, you know? It’s like my safe space, a place where nothing can touch me”.*

*(Samuel, male youth).*

### 3.4. Intergenerational Conflict

Participants reported difficulties engaging with their parents and feeling a great deal of pressure to conform and please them, while being pulled in different directions by the day-to-day Australian culture. A common issue raised by the participants was the expectations of parents and the general South Sudanese community that they do well in school and become either lawyers, doctors or engineers. If those career expectations were not met, they should pursue a business degree. While these lofty goals were attainable by some, many participants were aware that they did not have the academic results nor the desire to take on these careers. This created tension, with parents reporting that they believed that the young people were being disrespectful and rebellious. Many of the young people felt that their parents were more concerned with their standing within the South Sudanese community and the perception that a child studying to be a lawyer was a point of pride, whereas a child attending Technical and Further Education (TAFE) to become an electrician did not carry the level of community admiration. This often led to parents disregarding the young people’s career aspirations and creating further tension in a family dynamic that was already under stress.

*I don’t feel like I belong anywhere, not at school or home or anywhere. I started to feel like why I am here, like I thought my mom didn’t want me. She expects so much from me, I can’t do what she wants. No, it’s not fair sometimes. She thinks I am useless.*

*(Tut, male youth).*

The community forums that were held to validate the study results also enabled the young people and elders to talk to each other and share their perspectives. This allowed the young people to feel empowered in a public forum by being able to express and share how they felt. This resulted in a deeper understanding amongst all in attendance of each other’s concerns. The elders and parents were particularly open to hearing what the young people were experiencing especially as a disproportionate number of young people attempted and died by suicide at the time. Parents were desperate to know what to do to stop these tragedies from occurring.

*We don’t know why this happens [suicide]. We don’t know why they do it. So many kids kill themselves. They say nothing to us and then it happens. Why are they so unhappy, this is a good country, good opportunities? The kids don’t want to tell us what is wrong. Too many are dying, it’s too much.*

*(Dang, mother at community forum)*

### 3.5. The Development of the Community Empowerment Framework

**Figure 2:**
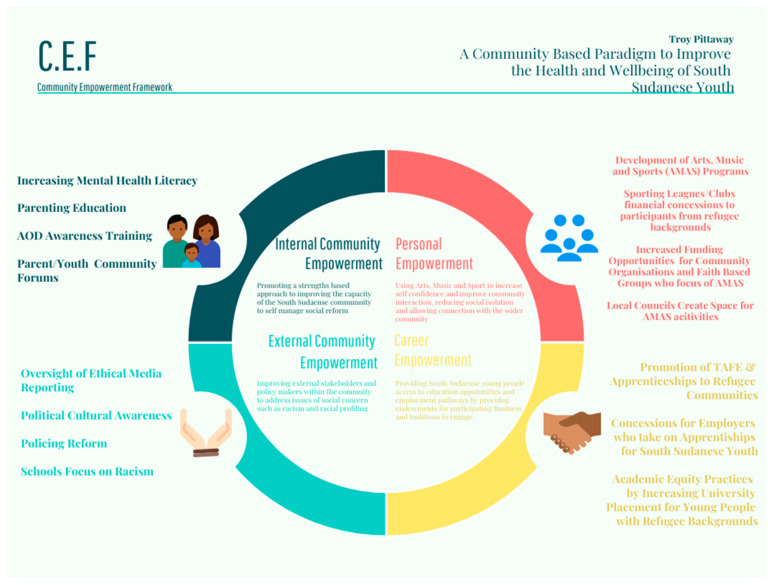
Community Empowerment Framework to Improve the Wellbeing of South Sudanese Youth (Alcohol and Other Drugs (AOD), Technical and Further Education (TAFE)).

An outcome of this research is the development of the Community Empowerment Framework (see Figure 2). The framework outlines the areas for implementation of interventions to address the key issues of concern identified by this research. Throughout the study, there were reports of coping strategies (e.g., playing sports), that could be replicated into programs, projects or public policy to improve the wellbeing of South Sudanese youth and their community. This framework expands upon the concepts of the Psychosocial Conceptual Framework [21] (See Figure 1) as it is tailored to the specific needs identified by the South Sudanese youth and community.

The Community Empowerment Framework has four domains (personal empowerment, career empowerment, internal community empowerment, external community empowerment) that operate at the individual, cultural community and the broader community (see Table 1). Overall, the framework aims to promote social inclusion, personal wellbeing and policy and practice reform. The four domains include, firstly, Personal Empowerment—using arts, music and sport to increase self-confidence and improve community interaction, reducing social isolation and allowing connection with the wider community. Secondly, Career Empowerment—enabling South Sudanese young people access to educational opportunities and employment pathways by providing endowments for participating businesses and institutions to engage. Thirdly, Internal Community Empowerment, which promotes a strengths-based approach to improving the capacity of the South Sudanese community to self-manage social reform and intergenerational conflict within their community. Finally, External Community Empowerment that aims to enable external stakeholders and policy makers within the community to address issues of social concern such as racism and racial profiling. Within these four domains, are specific strategies identified by the South Sudanese community during the interviews and forums, in which change can be achieved through intervention.

## 4. Discussion

This study drew on South Sudanese youth voices in Australia, where they experienced racism and intergenerational conflict and discussed their sense of belonging. A Community Empowerment Framework (CEF) was developed based on participants’ voices to identify the needs of South Sudanese youths. Four levels of empowerment strategies were discussed, including personal empowerment, career empowerment, internal community empowerment and external community empowerment. Collaboration between health and community workers, community elders and the youth could bridge issues of inter-generational contention, promoting the wellbeing of South Sudanese youths.

The proposed CEF outlined specific ways where empowerment leading to improved wellbeing can be achieved within the four levels. However, the strategies outlined are only small descriptions of how they can be applied. These descriptions are only guiding examples and starting points to initiate discussion. Implementation of the CEF could occur through community education programs and topic-focused workshop sessions. Services and programs need to recognize that each community has its own needs and strengths that programs need to be tailored to. A strength-based approach to empower youth in the co-design of practical strategies that meet their needs is critical.

The proposed CEF supplements current existing community development frameworks, especially with a focus that is built on South Sudanese youths. One of the existing community development frameworks is the Asset-Based Community Development (ABCD) model [37] This model focuses on six community assets that are crucial in building a community, which include individuals, cultures or stories, local economy, institutional, physical assets and associations [37]. Similar to our proposed CEF, the ABCD model also focuses on capacity building within the community, and building the community based on their current strengths [38]. This is also replicable in refugee and migrant-background communities [38]. However, there were some critiques against the ABCD model, which is vague and does not provide a clear and coherent approach for marginalized populations [39]. Hence, the proposed CEF aims to provide a more coherent model that centers around South Sudanese youths.

One example of using the proposed CEF would be the co-design of an educational program that supports positive interactions with South Sudanese youth and an improvement in the interaction between the police community engagement teams, allowing for a sensitive police force who understand the trauma faced by refugee youth. Policing reform is crucial, with a truth-telling and reconciliation investigation component into systematic racism. The police are a vital functioning part of society, creating safety for all citizens, if refugee youth feel threatened by those supposed to protect them, something significant needs to change.

It would be pertinent for politicians also to receive cultural sensitivity training, learn about different cultures, and have a standard implemented that stops racist and divisive language from being used by public representatives. This can increase their sense of belonging, within the social and political context [40]. The sense of belonging is important for all migrant groups in Australia, and acceptance plays a pivotal role in how migrant groups embrace their new culture [41].

Policy makers could create educational platforms for all schools to address racism in a systematic and deliberate way. This is not dissimilar to the School Support Program, which was developed in Victoria to address educational deficiencies for refugee students, and focused on celebrating cultural differences, which has the capacity to break down barriers that racism creates [42]. Promoting the strength and resilience of this community in co-developing programs and engagement to address these areas of concern is important.

Community organisations and faith-based groups can play a significant part in working with the South Sudanese community towards positive outcomes. This is possible through the networks and trust that they have within communities that might otherwise be resistant to intervention. Faith-based organisations are stakeholders in engaging young people through cultural and family connections, and therefore, make stakeholders ideal in being a platform to implement change. Faith-based organisations play a pivotal role, as there is a track record of positive outcomes for South Sudanese refugees who engage with developing social capital through going to church-run groups and youth groups [43]. This approach must be sensitive, with an awareness of fundamental elements associated with the religious practices of young people. It is important to engage the elders and leaders of the church and mosques to ensure that any development program is being conducted with the support of the faith group and is about fostering positive wellbeing for the young people without taking away any cultural or faith-based elements from the young people. It must be approached as a partnership, with clear shared goals.

Business and academic institutions could partner with the South Sudanese community, through Government incentives that focus on job creation and increased access to education. Central to this is the need to increase awareness around TAFE and apprenticeships as legitimate and valued career destinations. Amongst South Sudanese families there is a reticence to encourage young people to pursue these careers as they believe that they should be attaining higher levels of education regardless of whether the young person wants to do this or has the capacity to achieve it, this is not an uncommon issue amongst African refugee families in Australia [44]. Despite this, promoting various TAFE courses and apprenticeships could reduce the associated stigma around these careers within the South Sudanese community, resulting in more career options, less familial pressure and increased employment amongst this group of young people.

Encouraging businesses to employ young people after they obtain their qualifications is important, and giving financial incentives to businesses that employ young people from this background would help the transition from TAFE to employment. This would remove some of the barriers that are perceived to be in place by young people, particularly around racism in the workplace, with many giving up without trying due to this perception. Furthermore, universities could provide scholarships to allow students with refugee backgrounds to access courses. It is important to give credit to the incredible resilience of refugee communities facing the many obstacles that they have in acculturating and understanding that this resilience is easily transferable to good outcomes in education and employment—provided they are given an equitable chance to achieve positive outcomes.

## 5. Conclusions

As of 2024, South Sudan remains a country with a significant humanitarian crisis, conflict and inter-communal violence with a significant refugee and displaced population [45]. This case study explored some of the issues faced by South Sudanese young people as they contend with racism and intergenerational conflict and navigate life in their adopted culture. These experiences have led to the development of the CEF that is proposed in this article. It is our hope that the CEF will create a platform and an evolving framework to promote the wellbeing of South Sudanese youth. As a transformational tool that is aspirational in its design, the CEF aims to create the potential for both the South Sudanese community and the broader Australian community to engage and overcome the existing barriers to inclusion. Furthermore, this framework aims to support the South Sudanese community to address intergenerational conflict, while empowering the young people to feel valued. Active involvement of the South Sudanese community is critical to incorporate their strengths, needs and cultural understandings, with community and health organisations to ensure programs and activities are relevant, appropriate and meaningful. This, in turn, aims to empower the community to create the change that is needed to promote the wellbeing outcomes of South Sudanese young people.

## Figures and Tables

**Table 1:** The Community Empowerment Framework—identifying strategies.

Type of Empowerment	Pathway to Empowerment—Some Strategies
Personal Empowerment	Development of Arts, Music and Sports (AMAS) Programs: Community services and faith-based organisations should be informed of the social benefits of these types of programs. Focus on AMAS programs should be an available option for an early intervention response for community development and youth outreach services. Increased funding into AMAS Programs: Funding from local councils, states and federal governments should be increased to allow for the development and rollout of AMAS programs as a primary focus for marginalized youth, in particular migrant youth. Financial concession for sporting club participation: All sporting clubs should be allocated funding to support the participation of young people from South Sudanese backgrounds, as cost is a major prohibitive factor in participation in formal club activities. Local councils creating space to access AMAS activities: Local councils could be given funding incentives to create spaces for AMAS activities, like soccer fields, outdoor basketball courts and free space for art and equipment made available.
Career Empowerment	Promotion of Technical and Further Education (TAFE), Australia’s form of Technical and Vocational Education and Training and Apprenticeships as genuine career pathways: Educate the South Sudanese community of the importance of TAFE courses and apprenticeships, particularly in the potential of creating a small business and potential earning power of working in a trade. Corporate business to offer apprenticeships for South Sudanese youth: Funding for apprenticeships for companies that take on South Sudanese apprentices. Creating Academic equity by increasing university and TAFE placements for young people from refugee backgrounds. Increased university and TAFE placements and scholarships for South Sudanese youth who may not achieve the necessary high school results, accompanied by mentoring programs.
Internal Community Empowerment	Increasing mental health literacy: community forums run by trained South Sudanese social workers, bicultural workers, peer educators and public health professionals. Forums could be run in geographical locations where there are large cohorts of South Sudanese specific to ethnic groups i.e., Dinka, Nuer, Murle and many other ethnic groups or together based on community preference. Parenting education: Parenting groups run by legal practitioners, social workers and public health workers, within South Sudanese background, outlining the legal system. This could also include discussing parenting styles within Australia and South Sudan, illustrating the ‘nuclear family’ and its impact on Australian social life, and how traditional African parenting differs. AOD awareness: Trained AOD workers or social workers within the South Sudanese community could facilitate education groups to support parents, elders and young people about the impact of drugs and alcohol in an informative, harm reduction setting. Parent/youth community forums: Facilitated groups with elders and parents of youth run by leaders and elders within the community to support the exchange of ideas and initiate opening communications between the generations.
External Community Empowerment	Political cultural awareness: Programs developed within local councils, state government and federal government on cultural diversity, to increase empathy, social justice and human rights literacy and understanding of the refugee experience, within Australia. Policing reform: Police to participate in professional development and cultural awareness to work better with various ethnic groups and recognize cultural reactions and triggers, such as physical deference to authority, i.e., not looking the police in the eye when talking, and other potential misunderstandings. This should be firmly integrated into how policing is practised, which would result in a trauma-informed police workforce. Oversight of ethical media reporting: Improved oversight into the content and language used by the media monitored by media watchdogs. Anti-racism education in schools: Co-design, funding and roll-out of programs to improve whole-of-school understanding of racism and how to address it.

## Data Availability

The data used and analyzed for this study are available from the corresponding author upon reasonable request.
